# Supplementary material for: Tree-rings reveal two strong solar proton events in 7176 and 5259 BCE
Source: Nat Commun. 2022 Mar 7;13:1196. doi: 10.1038/s41467-022-28804-9 (PMC8901681; doi:10.1038/s41467-022-28804-9)
Supplement: Supplementary file 1 — Supplementary Information [file 41467_2022_28804_MOESM1_ESM.pdf]

Supplementary Information

S1 Dendrochronology

Dendrochronological dating of waterlogged oak samples from the river Main, Germany (M49 and M234)

The subfossil oak (*Quercus* sp.) samples, M49 and M234, used for this study were collected from waterlogged trunks discovered during gravel quarrying along the River Main in the 1970s and 1980s. Samples were obtained from a veneer mill near Bamberg, which obtained timber from a series of gravel pits along an 80 km stretch of the River Main between Schweinfurt (50.05N; 10.24E) and Lichtenfels (50.15N; 11.05E). The provenance of these particular timbers is not known exactly.

These samples were selected for inclusion in this study because the growth rings of the trees were > 1mm in width, and the samples were without distortion caused by growth anomalies or knots that would make the dissection of the single tree-rings for radiocarbon dating difficult.

The wet samples from the gravel pits were prepared using a scalpel blade to remove the rough wood from the sample surface to expose the tree-ring pattern. The ring-widths were then measured to a precision of 1/100 mm using a micro-computer based travelling stage.

The individual ring-width series for M234 was cross-dated against the Göttingen South German Oak Chronology[1], and was further compared with the Hohenheim Southern German Oak chronology[2], which over this time period was constructed separately from the Göttingen master chronology. The correlation values obtained are given in Table S1.1. The individual ring-width series for M49 were cross-dated against the Hohenheim Southern German Oak Chronology. The correlation values obtained are also given in Table S1.1. Figure S1.1 shows the ring-width series of M49 and M234 on a logarithmic scale against the Hohenheim master chronology.

Table S1.1: *overlaps (years) / Gleichläufigkeit (%) / t-values (Ballie and Pilcher 1973) between M49, M234, the Göttingen South German Riverine Oak Chronology and the Hohenheim South German Oak Chronology.*

	Hohenheim South German oak	M49	M234
Göttingen South German oak	8797 / 78 / 91.5		
Göttingen South German oak (without M234)			133 / 67 / 6.5
Hohenheim Southern German oak		118 / 69 / 10.0	133 / 69 / 8.5
M49			39 / 66 /3.0

The good visual matching of the ring-width series (Figure S1.1), along with the correlation values obtained (Table S1.1) dates the 118-ring series, M49, as spanning 7276–7159 BCE and the 133-ring series, M234, as spanning 7197–7065 BCE. The calendar dates provided are historic dates BCE without the year zero.

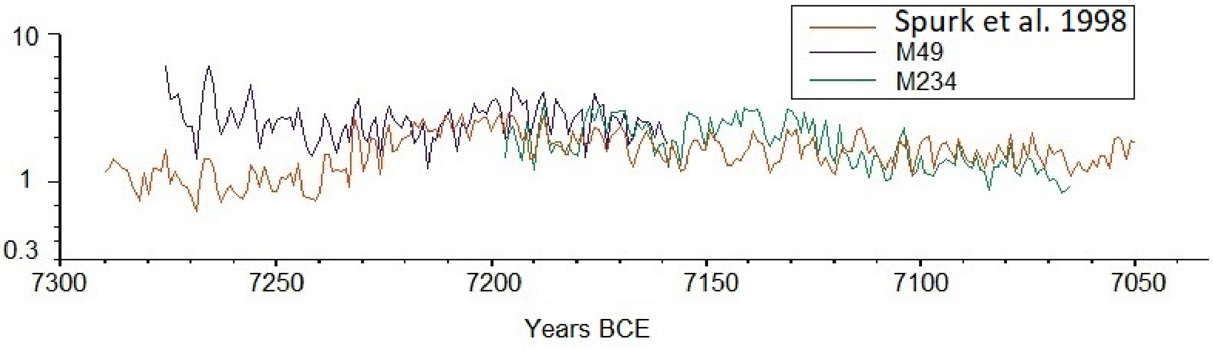

Figure S1.1: *(a) Ring-width series of M49 and M234 on a logarithmic scale against the Hohenheim master chronology (Friedrich et al. 2004).*

Timbers from the Main river gravels were originally waterlogged, but have subsequently been allowed to dry out naturally. The remaining parts of the samples are stored in the Department of Palynology and Climate Dynamics, Albrect-von-Haller Institute for Plant Sciences, Georg-August-Universität-Göttingen, Wilhelmsplatz 1, 37073 Göttingen, Germany. Ring-width data for the two tree slices sampled for radiocarbon dating are provided in Table S1.2.

Dissection for radiocarbon dating was undertaken by Alison Arnold and Robert Howard of the Nottingham Tree-ring Dating Laboratory. The samples to be used were cleaned with a razor blade and the annual growth rings marked. At this stage it became apparent that 22 rings from the centre of M232, and two from the outside, had crumbled during storage, and only the rings spanning 7175–7067 BC were available for dissection.

The annual growth rings were split from the bulk sample using a scapel blade. Whole ring samples, including both earlywood and latewood, were provided to the ETH laboratory from M49 (7276–7160 BCE) and M232 (7175–7067 BCE). Replicate whole ring samples, including both earlywood and latewood, were provided to the Bristol laboratory from M49 (7181–7160 BCE). Further replicate whole ring samples from M49 (n=29) and M234 (n=13) have been archived by Historic England.

The annual growth rings from M49 (7178–7175 BCE) were further divided using a scapel into separate samples comprising earlywood only and latewood only, which were provided to both the ETH and Bristol laboratories.

Finally, the annual growth ring for 7177 BCE from M49 was split into early and late earlywood (EW1 and EW2), and early and late latewood (LW1 and LW2) and the samples supplied to the ETH laboratory.

Table S1.2: *Ring-width data for the timbers which provided single-ring samples for radiocarbon calibration (Heidelberg format)*

HEADER:
KeyCode=SM001D_0
DataFormat=Tree
Project=S
Country=S
LocationCharacteristics=M
SiteCode=00
TreeNo=1D
CoreNo=0
SeriesType=Single curve
StatisticalDataTreatment=O
DataType=Ringwidth
Unit=mm
Dated=Dated
Length=118
DateBegin=-7276
DateEnd=-7159
Species=Qusp
Location=M49
PersId=HL
LastRevisionDate=01.04.90
MissingRingsBefore=25
DeltaMissingRingsBefore=10
MissingRingsAfter=20

DeltaMissingRingsAfter=.
Comment=SM000490.!OJ/_
DATA:Single

587	359	361	380	268	233	231	141	250	447
586	438	272	203	234	306	271	225	275	353
442	278	165	237	260	208	252	265	257	266
221	309	213	160	146	162	184	279	243	190
155	203	239	179	291	360	199	180	226	265
148	249	316	268	241	226	275	250	176	195
243	121	195	185	235	253	298	245	156	242
224	245	333	283	293	281	336	359	315	189
201	417	392	323	345	197	252	338	395	247
206	353	311	279	207	254	277	304	143	223
380	318	326	161	275	267	238	297	171	193
270	248	247	240	197	251	196	171	0	0

HEADER:
KeyCode=SM006I_0
DataFormat=Tree
Project=S
Country=S
LocationCharacteristics=M
SiteCode=00
TreeNo=6I
CoreNo=0
SeriesType=Single curve
StatisticalDataTreatment=O
DataType=Ringwidth
Unit=mm
Dated=Dated
Length=133
DateBegin=-7197
DateEnd=-7065
Species=Qusp
Location=M 234
PersId=HL
LastRevisionDate=01.04.90
MissingRingsBefore=0
DeltaMissingRingsBefore=0
MissingRingsAfter=0
DeltaMissingRingsAfter=0
Comment=SM002340.!OJ/ Probe fehlt
DATA:Single

144	207	230	177	135	250	202	119	231	331
184	153	171	169	155	173	155	146	218	261
315	254	313	244	208	285	283	295	293	224
192	242	222	241	223	146	172	177	123	184
182	128	241	288	275	267	206	168	268	196
229	262	241	238	296	230	306	291	291	298
242	190	167	201	187	239	302	294	269	208
284	211	261	150	188	252	153	129	234	158
149	121	125	157	145	105	146	145	119	99
102	154	192	226	145	108	116	148	112	110
106	126	129	141	132	126	183	139	126	121
146	118	116	87	124	125	136	124	175	114
108	127	144	135	111	117	120	100	101	97
82	87	92	0	0	0	0	0	0	0

Dendrochronological dating of the bog oak samples from Derrymacfall, Co. Armagh, Northern Ireland (Q 2729 and Q 2750)

In July 1977, slices were collected from seventy-one bog oak (*Quercus* sp.) timbers from the townland of Derrymacfall, just south of Lough Neagh, Co. Armagh, Northern Ireland (54° 28'N, 6° 28'W).

The wet samples were prepared using a scalpel blade to remove the rough wood from the surface of the sample and to expose the tree-ring pattern. If the wood sample was soft or the ring pattern needed to be clarified, a razor blade was used. Finely ground chalk was rubbed onto the prepared surface to define the annual tree-ring boundaries more clearly for measurement. The ring-widths were measured to a precision of 1/50mm using a travelling stage between November 1979 and September 1980. Sometimes more than one radius was measured for an individual sample (e.g. Q2737A/B), and so some ring series are a mean of two or more measurement series on the same sample.

The ring-width series of nine of these samples as well as those of two samples from Loughmagarry, Co. Antrim (Q2527 and Q2528; 54° 52'N, 6° 17'W) were included in the Derrymacfall 2 master chronology (Table S1.3). The construction of the chronology followed the procedures described by Baillie[1]. Samples with the best correlation values were combined to form sub-site masters. These were then used to date others samples. The other dated samples were incorporated into a new sub-site master until a coherent master chronology was formed. The individual samples were then compared with each other giving the correlation values presented in the *t*-value matrix (Table S1.4). The correlation *t*-values are based on CROS84[2]. At this time, the Derrymacfall 2 master chronology could not be dated by dendrochronology.

Table S1.3: *Details of the cross-matched samples from the Derrymacfall 2 master chronology (?h/s = outermost measured ring is possible heartwood/sapwood boundary)*

Sample	Rings	Sapwood Rings	Absolute Dating (BCE)
Derrymacfall, Co Armagh			
Q2682	159	No sapwood	BCE 5429 – 5271
Q2717	160	No sapwood	BCE 5446 – 5287
Q2720	100	No sapwood	BCE 5353 – 5254
Q2729	181	24	BCE 5356 – 5176
Q2734	91	No sapwood	BCE 5318 – 5228
Q2737M	176	No sapwood	BCE 5457 – 5282
Q2750	267	?h/s	BCE 5428 – 5162
Q2779	190	No sapwood	BCE 5389 – 5200
Q2781	139	No sapwood	BCE 5419 – 5281
Loughmagarry, Co Antrim			
Q2527	104	No sapwood	BCE 5378 – 5275
Q2528	133	No sapwood	BCE 5392 – 5260

In 1989 re-measurement of bog oak samples from a number of sites was undertaken. This work produced local site chronologies which cross-dated both with the Derrymacfall 2 master chronology and with the original Belfast Long Chronology (BLC5000). When the Derrymacfall 2 master chronology was compared with these chronologies extremely significant and consistent correlation values were found with the Tullyroan 10 and the Motorway 2 chronology.

Table S1.4: *t-value matrix for ring-width series in the Derrymacfall 2 master chronology (- = no correlation value; nsm = non-significant match; nh = not highest match; * = significant match; ** = very significant match; *** = extremely significant match)*

QUB ID	Q2717	Q2682	Q2750	Q2781	Q2528	Q2779	Q2527	Q2729	Q2720	Q2734
Q2737M	2.33nh	6.59***	2.87nh	3.55nsm	2.96nm	5.32***	2.82nh	3.47nsm	4.02nsm	-
Q2717	-	4.18nsm	3.67nsm	3.40nsm	1.86nh	6.41***	0.38nm	3.33nsm	5.38***	-
Q2682	-	-	5.46***	6.94***	2.62nh	7.08***	1.60nh	3.05nsm	5.09***	-
Q2750	-	-	-	3.97nsm	4.51nsm	5.66***	3.04nh	5.87***	7.44***	3.80nsm
Q2781	-	-	-	-	3.55nsm	6.51***	3.20nsm	2.48nh	4.28*	-
Q2528	-	-	-	-	-	3.92*	7.31***	2.97vwm	2.45nh	-
Q2779	-	-	-	-	-	-	2.49nh	3.58nsm	6.88***	2.81nh
Q2527	-	-	-	-	-	-	-	2.68nh	1.29nh	-
Q2729	-	-	-	-	-	-	-	-	4.24*	2.98nsm
Q2720	-	-	-	-	-	-	-	-	-	3.29nsm

The Derrymacfall 2 master chronology consists of eleven samples (Figure S1.2). It is 296 years in length and dates from BCE 5457 to BCE 5162. Table S1.5 gives the correlation values of this master chronology with other site chronologies from Ireland. Only correlation values over t = 4 are included in the table. The correlation t-values are based on CROS84[2].

Table S1.5: *Correlation values for the 296-year Derrymacfall 2 master chronology with other site and regional chronologies (*** = extremely significant match ; ** = very significant match)*

Site chronology	Start and end date of chronology	Correlation value with Derrymacfall 2
Tullyroan 10, Co Armagh	5334BCE to 4680BCE	t = 8.39***
Motorway 2, Co Armagh	5474BCE to 5228BCE	t = 5.75***
Killyleagh 1, Co Down	5236BCE to 4829BCE	t = 4.62**

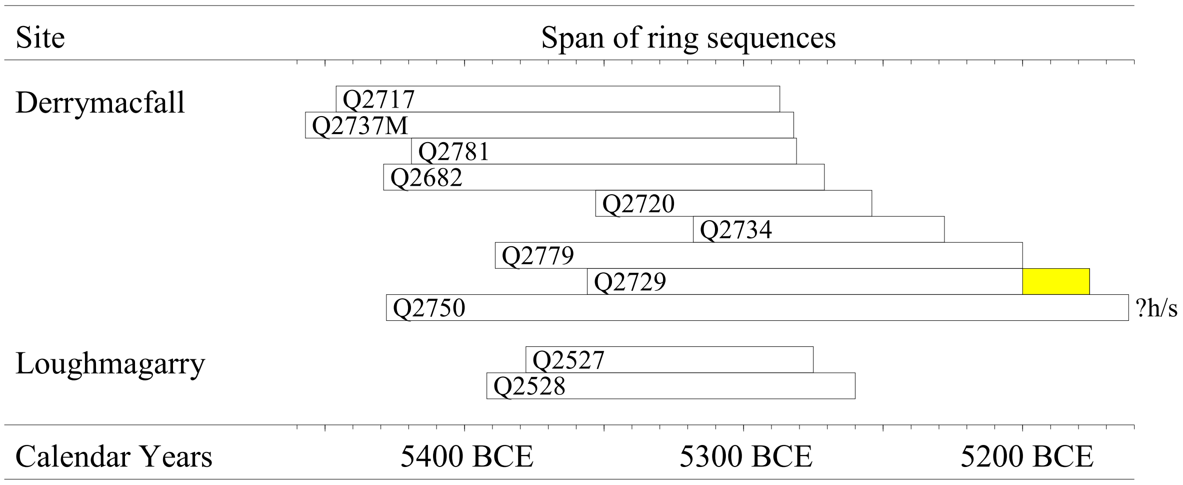

Figure S1.2: *Bar diagram showing the relative positions of the dated samples. White bar = heartwood; yellow bar = sapwood;?h/s = outermost measured ring is possible heartwood/sapwood boundary*

Timbers from Derrymacfall were originally waterlogged, but subsequently have been allowed to dry out naturally. The remainder of the samples are stored in the Dendrochronology Laboratory, School of Natural and Built Environment, Queen's University, Belfast. Ring-width data for all the measured samples from Derrymacfall can be found at http://www.chrono.qub.ac.uk/bennett/dendro_data/dendro.html.

Two samples from the Derrymacfall 2 site chronology, Q2729 and Q2750 (Table S1.6), have been used to supply single-year samples for radiocarbon calibration. For the selected period, the trees with the widest annual growth rings were chosen, so that extracting the individual annual growth rings is as easy as possible. The samples to be used were cleaned with a razor blade and the annual growth rings marked. The annual growth rings were split from the bulk sample using a scalpel bade and the early wood growth was removed using a scalpel. This processing means that the wood samples provided only have the latewood part of the annual growth ring.

Sample Q2729 was used to supply samples from 5300 BCE to 5211 BCE inclusive, and sample Q2750 was used to supply samples from 5210 BCE to 5175 BCE inclusive. Those single-ring samples that weighed more than 120mg were divided further using a scalpel to supply duplicate samples of 90 single rings for inter-laboratory replication.

Table S1.6: *Ring-width data for the timbers which provided single-ring samples for radiocarbon calibration (Heidelberg format)*

HEADER:
Key Code = Q2729
Data Format = Tree
Location = Derrymacfall, Co. Armagh
Length = 181
Date Begin = 5356BC
Date End = 5176BC
Dated = Dated
Comment = EDH 17-June-1980 Close to centre 24 sapwood rings not complete
Key No = Q2729
Project = Derrymacfall, Co. Armagh

Species = QUSP
Tree No = Q2729
Pith = -
WaldKante = ---
Sapwood Rings = 24
Unit = 1/50 mm
Data Type = Ring width
Series Type = Single curve
Series Start = Ring width
Series End = Ring width
Global Math Comment Count = 0
Image Count = 0
Comment Count = 0
Bibliography Count = 0
DATA: Single

56	46	56	76	66	84	104	75	77	73
41	51	66	73	78	76	96	91	78	78
100	71	73	59	58	62	53	43	84	71
75	95	74	139	126	144	131	140	108	88
75	85	53	36	42	47	73	73	67	70
110	132	212	158	177	126	107	113	85	99
80	98	106	90	92	92	97	103	150	157
113	130	127	81	85	96	95	94	82	100
79	94	81	99	105	126	118	106	94	67
65	49	63	48	61	52	44	43	64	55
63	51	69	58	58	62	63	61	61	63
68	91	65	60	65	75	54	53	53	53
43	44	32	30	29	27	36	29	29	34
39	98	61	72	28	26	26	31	39	34
46	45	64	69	49	51	55	46	35	30
29	25	29	29	30	46	45	34	44	31
36	31	37	33	31	38	37	36	23	28
30	31	34	60	74	60	43	28	31	34
19									

HEADER:
Key Code = Q2750
Data Format = Tree
Location = Derrymacfall, Co. Armagh
Length = 267
Date Begin = 5428BC
Date End = 5162BC
Dated = Dated
Comment = EDH 9-Aug-1980 Close to centre no sapwood possible H/S boundary
Key No = Q2750
Project = Derrymacfall, Co. Armagh
Species = QUSP
Tree No = Q2750
Pith = -
WaldKante = ---
Unit = 1/50 mm
Data Type = Ring width
Series Type = Single curve
Series Start = Ring width
Series End = Ring width
Global Math Comment Count = 0
Image Count = 0
Comment Count = 0

Bibliography Count = 0
DATA: Single

15	16	17	5	14	4	19	15	12	11
11	9	19	21	25	22	14	7	26	16
8	21	33	40	73	48	32	23	17	26
23	50	69	76	28	35	48	49	44	57
37	28	23	85	95	43	48	49	77	84
127	164	157	105	97	66	67	86	101	87
41	25	31	42	21	13	17	13	25	19
16	13	17	19	18	22	17	33	40	38
33	16	8	13	12	18	16	25	40	42
18	25	24	11	10	8	12	11	7	9
16	15	17	13	11	16	18	19	19	32
28	31	33	37	19	10	12	15	30	29
28	33	40	47	30	38	59	51	28	25
18	18	19	29	59	22	16	23	28	39
66	45	25	23	26	46	39	44	80	66
40	62	64	48	44	50	43	21	25	18
21	29	37	27	55	32	45	41	32	24
60	102	84	42	57	41	29	33	46	37
28	46	27	26	30	34	20	45	30	31
27	22	15	36	32	33	29	40	60	14
21	24	56	50	35	26	17	12	6	13
11	9	7	9	17	15	25	31	23	20
21	24	30	47	33	35	34	60	47	40
27	28	18	22	48	62	79	60	58	51
29	46	37	56	48	46	52	59	49	16
23	26	19	13	23	29	22	28	12	18
13	21	28	38	44	47	33			

Bristlecone Pine

Two bristlecone pine (Pinus Longaeva D.K. Bailey) samples were used in this study. For the 5259 BCE event, sample MWK80-101 was selected from the calendar dated master Methuselah Walk bristlecone pine chronology[3]. The sample derives from a single *Pinus longaeva* D.K.Bailey tree which grew at the Methuselah Walk site (MWK) in the White Mountains of California in the western USA. The remnant tree was preserved in a dry, high altitude (c.2900 m a.s.l.) mountain environment. The full tree sampled had 747 rings with no pith, bark or waney edge present. Standard convention for bristlecone dating is via the use of the astronomical calendar (AD/BC with 0) for which details are provided in Ferguson *et al.*[3]. Chronologies are constructed using a combination of graphical skeleton plotting[4], and / or tree-ring measurement, and then statistical verification using COFECHA[5]. For MWK80-101, calendar dates were assigned relative to the master MWK chronology and the sample was lagged successively by 25 years using COFECHA12K version 6.06P. The sample spans -5821 to -5075 (747 years, 30 50-yr segments, 0 flags and demonstrates excellent correlation with the master MWK chronology; r = 0.770). The COFECHA outputs are provided below in AD/BC with 0. Two sub-samples of MWK80-101 were marked with dated years and sampled from the cross-dated main sample. The sub-samples were cut to be c. 5 mm thick relative to the transverse section. The upper and lower surface were lightly sanded (preserving original pin-holes) but removing any surface contamination and excess resins which come up to the sample surface over time. The upper, lower and radial surfaces were clearly marked with the pin-holes and measurements re-checked prior to dissection. Dissection was carried out using the astronomical calendar (AD/BC with 0) under times 20 magnification using a binocular microscope and steel disecting blade. The same blade was used to remove any residual contamination possibilities (pinholes, any residual cells from the previously dissected year of interest) prior to sampling each seperate year of interest. Samples were dissected

working from youngest to oldest and packed in pre-marked foil packets. We note that a correction of 2 years was applied to Wes Fergusons original 1966 CE dating of this part of the MWK chronology due to an observation by Lamarche and Harlan in 1973[6] that Ferguson had incorrectly added what he thought were two likely missing rings to his chronology in -2141 and -2680. Chris Baisan and Mathew Salzer have since demonstrated that the first of these was a clerical error and the second a false ring, placed in 1966 by Ferguson, so both these erroneous years are removed from the master chronology used in this study.

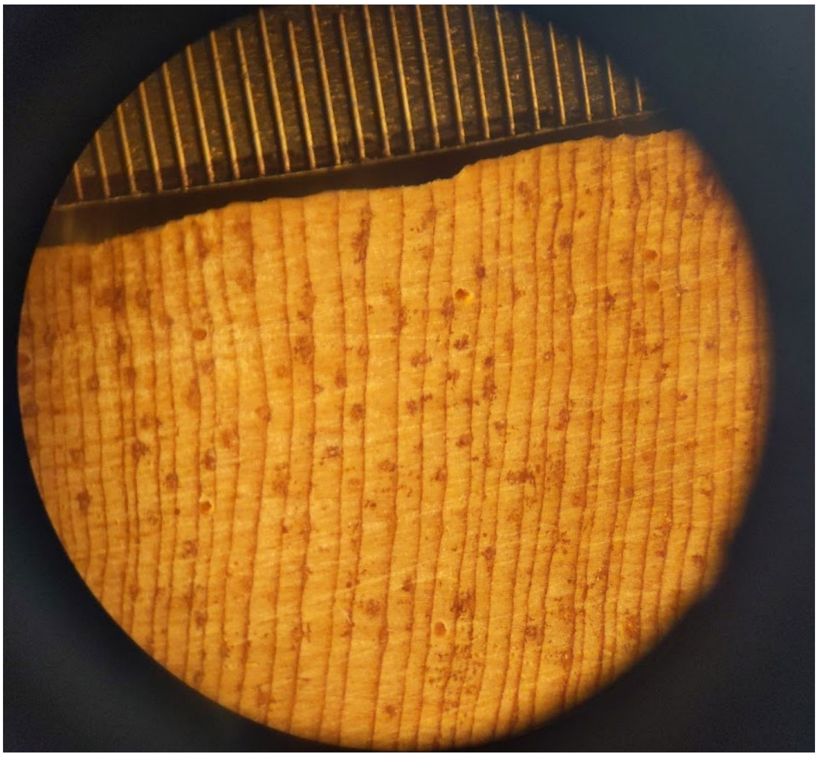

Figure S1.3: Dissected rings of sample MWK80-101 with 1mm ruler for scale. Double pinhole is the year -5250.

COFECHA12K version 6.06P output showing correlations of individual 50-year dated segments, lagged 25 years from MWK-80-101:

Flags: A = correlation under .3281 but highest as dated; B = correlation higher at other than dated position

-5700 to -5176

2	03-876	-6261-5548	.63	.49	.79	.81	.64	.65														
33	67-035	-5553-4714						.57	.57	.62	.61	.58	.52	.65	.78	.78	.77	.62	.67	.79	.65	.62
38	69-202	-6223-5096	.75	.65	.58	.58	.57	.74	.79	.78	.81	.74	.65	.77	.86	.80	.80	.75	.80	.89	.78	.77
40	70-020	-5460-4708										.67	.68	.72	.80	.76	.79	.68	.72	.89	.80	.78
43	70-043	-6377-5408	.62	.58	.59	.59	.55	.52	.65	.71	.78	.80	.59									
44	70-046	-6066-5008	.70	.71	.76	.65	.52	.63	.62	.70	.77	.68	.51	.52	.55	.69	.81	.61	.53	.63	.70	.63
46	70-176	-6045-5039	.67	.62	.73	.79	.64	.64	.66	.69	.64	.71	.70	.53	.67	.75	.83	.56	.57	.70	.65	.61

48	71-059	-5922-5302	.76	.82	.84	.87	.73	.55	.60	.79	.90	.79	.69	.61	.70	.37	.44					
49	71-060	-5880-5324	.62	.50	.62	.55	.41	.59	.43	.53	.71	.70	.58	.63	.83	.63	.62					
50	71-061	-5419-4931												.81	.80	.82	.80	.75	.86	.90	.85	.88
53	71-161	-5340-4819															.59	.64	.77	.85	.73	.69
55	72-104	-5722-4960	.69	.74	.79	.78	.67	.65	.53	.63	.81	.70	.58	.77	.87	.57	.67	.85	.86	.78	.62	.67
56	72-175	-6059-5080	.58	.68	.76	.84	.74	.68	.80	.85	.86	.79	.79	.85	.84	.69	.75	.79	.86	.89	.82	.82
61	77-104	-5747-4487	.68	.78	.84	.87	.82	.67	.53	.63	.76	.74	.66	.74	.82	.64	.74	.85	.86	.89	.77	.73
72	77-151	-5230-4748																			.67	.74
76	77-162	-5673-4821		.70	.82	.86	.80	.77	.77	.79	.80	.76	.79	.80	.84	.84	.91	.93	.88	.85	.79	.78
83	78-026	-6065-5445	.75	.57	.72	.90	.67	.63	.70	.72	.84	.81										
89	78-075	-5598-4575					.73	.66	.70	.73	.80	.74	.66	.65	.80	.81	.84	.84	.70	.74	.81	.81
92	79-026	-6166-5458	.70	.64	.84	.84	.76	.57	.56	.68	.80											
95	79-058	-6012-4899	.81	.78	.83	.87	.75	.64	.63	.68	.76	.70	.78	.81	.76	.75	.85	.88	.89	.87	.81	.86
104	79-085	-5377-4989													.67	.66	.74	.63	.66	.84	.52	.52
119	79-136	-6245-5448	.77	.75	.71	.75	.69	.54	.58	.70	.80	.80										
125	***80-101***	***-5821-5075***	***.78***	***.66***	***.70***	***.77***	***.73***	***.79***	***.69***	***.58***	***.75***	***.74***	***.68***	***.85***	***.91***	***.74***	***.69***	***.74***	***.70***	***.79***	***.75***	***.74***
127	80-103	-5361-4491														.81	.82	.83	.77	.77	.82	.78
133	80-110	-6022-4238	.74	.65	.78	.84	.64	.60	.78	.78	.77	.80	.80	.77	.79	.84	.83	.81	.80	.76	.72	.79
136	80-116	-5685-2822	.75	.68	.73	.81	.80	.81	.84	.79	.75	.83	.77	.81	.93	.84	.83	.72	.83	.92	.82	.76
184	93-031	-6133-5618	.62	.70	.74																	
234	XX-001	-6056-3998	.73	.61	.75	.84	.84	.64	.52	.60	.63	.65	.66	.69	.71	.57	.54	.67	.74	.73	.67	.68
235	XX-002	-5323-3562																.69	.80	.81	.76	.65
Av segment correlation			.70	.67	.75	.78	.69	.64	.65	.70	.77	.74	.67	.72	.78	.72	.75	.74	.76	.81	.74	.73

-5200 to -5051

33	67-035	-5553-4714	.69	.67	.75	.75	.64
38	69-202	-6223-5096	.87	.81	.78	.73	
40	70-020	-5460-4708	.79	.71	.75	.82	.79
44	70-046	-6066-5008	.44	.49	.51	.49	.73
46	70-176	-6045-5039	.53	.53	.62	.74	.83
50	71-061	-5419-4931	.79	.74	.79	.90	.75
53	71-161	-5340-4819	.63	.65	.77	.82	.59
55	72-104	-5722-4960	.75	.67	.71	.77	.78
56	72-175	-6059-5080	.90	.91	.90	.91	
61	77-104	-5747-4487	.87	.79	.82	.86	.80

72	77-151	-5230-4748	.79	.71	.71	.78	.63
74	77-158	-5199-4495	.62	.65	.67	.67	.59
76	77-162	-5673-4821	.82	.85	.79	.67	.61
89	78-075	-5598-4575	.78	.81	.82	.66	.53
93	79-050	-5198-4891	.64	.81	.81	.67	.74
94	79-057	-5169-4706		.80	.80	.86	.85
95	79-058	-6012-4899	.83	.75	.81	.85	.77
98	79-075	-5095-4163					.83
101	79-082	-5077-4733					.84
104	79-085	-5377-4989	.62	.65	.76	.80	.72
107	79-091	-5102-4306				.64	.64
125	80-101	-5821-5075	.78	.83	.88	.91	.92
127	80-103	-5361-4491	.76	.70	.75	.82	.83
133	80-110	-6022-4238	.78	.81	.84	.84	.70

-5200 to -5051 (cont.)

136	80-116	-5685-2822	.80	.78	.79	.86	.85
234	XX-001	-6056-3998	.61	.57	.60	.55	.66
235	XX-002	-5323-3562	.70	.74	.77	.87	.84
Av segment correlation			.73	.73	.76	.77	.74

Samples from the MWK chronology show strong cross-matching with other independently developed bristlecone pine chronologies (across 5750 years) from Sheep Mountain and Indian Garden. These chronologies are available at: https://catalog.data.gov/dataset/international-tree-ring-data-bank-itrdb and further details are provided in Salzer and Hughes, 2007[7], 2010[8], Salzer et al 2009[9], 2014[10], Kipfmueller, 2010[11].

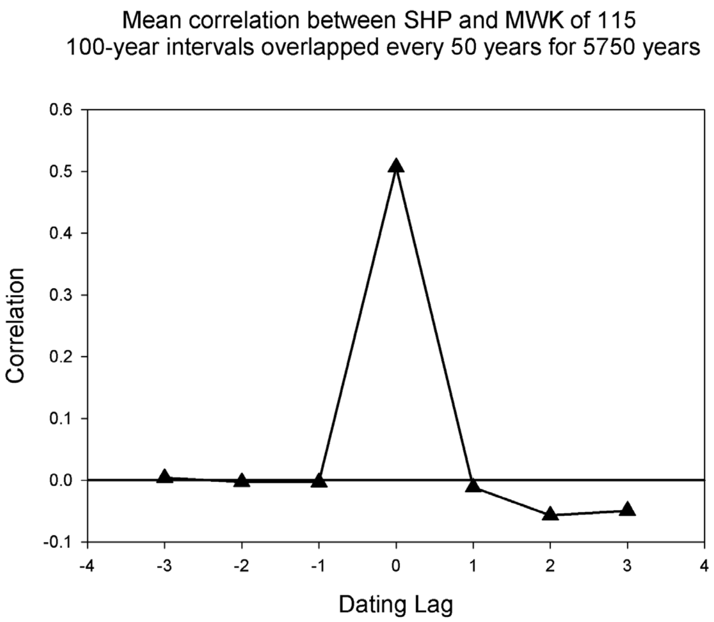

Tree-ring width measurements of the dissected subsamples used in this study are provided below:

Tree ID	Astronomical calendar (AD/BC with 0)	BCE	BP	80-101B SubSample1 Ringwidth	80-101B SubSample2 Ringwidth
MWK-80-101B	-5270	5271	7220	0.408	0.599
MWK-80-101B	-5269	5270	7219	0.464	0.62
MWK-80-101B	-5268	5269	7218	0.644	0.831
MWK-80-101B	-5267	5268	7217	0.634	0.83
MWK-80-101B	-5266	5267	7216	0.526	0.656
MWK-80-101B	-5265	5266	7215	0.704	0.909
MWK-80-101B	-5264	5265	7214	0.502	0.698
MWK-80-101B	-5263	5264	7213	0.76	0.999
MWK-80-101B	-5262	5263	7212	0.628	0.811
MWK-80-101B	-5261	5262	7211	0.47	0.58
MWK-80-101B	-5260	5261	7210	0.61	0.94
MWK-80-101B	-5259	5260	7209	0.29	0.47
MWK-80-101B	-5258	5259	7208	0.342	0.501
MWK-80-101B	-5257	5258	7207	0.506	0.643
MWK-80-101B	-5256	5257	7206	0.414	0.471
MWK-80-101B	-5255	5256	7205	0.434	0.52
MWK-80-101B	-5254	5255	7204	0.644	0.856

MWK-80-101B	-5253	5254	7203	0.232	0.496
MWK-80-101B	-5252	5253	7202	0.498	0.687
MWK-80-101B	-5251	5252	7201	0.278	0.399
MWK-80-101B	-5250	5251	7200	0.338	0.489

For the 7176 BCE event, sample MWK85-051 from the non-calendar dated portion of the Methuselah Walk bristlecone pine master chronology was selected. This sample derives from a single *Pinus longaeva* D.K.Bailey tree which grew at the Methuselah Walk site (MWK) in the White Mountains of California in the western USA. The remnant tree was preserved in a dry, high altitude mountain environment. The full tree sampled had 474 rings with no pith, bark or waney edge present. This tree is not part of the existing MWK master chronology but was placed in time by a possible dendrochronological match backed by limited, course resolution radiocarbon dating[12] (Salzer *et al*. 2019). The sample was selected with the aim of testing the possible placement and using annual ^{14}C to firmly connect this sample with the MWK master tree-ring chronology, extending this sequence securley back in time. Following dissection of the first sub-sample at annual resolution it became clear that an adjustment of around 40 years was required to this dating in order to detect the 7176 BCE event. A second sampling was carried out, with the floating astronomical calendar (AD/BC with 0) years adjusted to be 40 years older. The annual ^{14}C analysis of the second subsample detected the 7176 BCE event, indicating a further 1-year adjustment was necessary to align the floating bristlecone pine series with the other, annual ^{14}C measurements on calendar dated trees. This extends the continuous sequence for MWK over 10,398 years.

The dissected years and associated sub-sample ringwidths are provided in the table below:

Dating A (AD/BC with 0) NOT CALENDAR SECURE – floating placement: course resolution radiocarbon	Dating A (BP) NOT CALENDAR SECURE – floating placement: course resolution radiocarbon	MWK-85-051B Subsample1 Ringwidth	Dating B (AD/BC with 0) NOT CALENDAR SECURE – floating placement: higher resolution radiocarbon	Dating B (BP) NOT CALENDAR SECURE – floating placement: higher resolution radiocarbon	MWK-85-051B SubSample2 Ringwidth
-7186	9136	402	-7160	9150	0.682
-7185	9135	260	-7159	9149	1.036
-7184	9134	490	-7158	9148	0.684
-7183	9133	602	-7157	9147	0.472
-7182	9132	656	-7156	9146	0.614
-7181	9131	596	-7155	9145	0.5
-7180	9130	720	-7154	9144	0.194

-7179	9129	760	-7153	9143	0.066
-7178	9128	640	-7152	9142	0
-7177	9127	592	-7151	9141	0.25
-7176	9126	496	-7150	9140	0.186
-7175	9125	574	-7149	9139	0.398
-7174	9124	388	-7148	9138	0.646
-7173	9123	796	-7147	9137	0.498
-7172	9122	996	-7146	9136	0.54
-7171	9121	878	-7145	9135	1.476
-7170	9120	690	-7144	9134	0.408
-7169	9119	556	-7143	9133	0.32
-7168	9118	804	-7142	9132	0.218
-7167	9117	630	-7141	9131	0.526
-7166	9116	540	-7140	9130	0.898
-7165	9115	808	-7139	9129	0.61
-7164	9114	732	-7138	9128	1
-7163	9113	924	-7137	9127	1.308
-7162	9112	338	-7136	9126	0.85
-7161	9111	446	-7135	9125	0.346
-7160	9110	884	-7134	9124	0.448
			-7133	9123	0.658
			-7132	9122	0.92
			-7131	9121	1.118
			-7130	9120	1.48
			-7129	9119	0.894
			-7128	9118	0.49
			-7127	9117	0.738
			-7126	9116	0.78
			-7125	9115	0.884
			-7124	9114	0.974
			-7123	9113	1.018

-7122	9112	0.484
-7121	9111	0.572
-7120	9110	0.482
-7119	9109	0.45
-7118	9108	0.402
-7117	9107	0.602
-7116	9106	0.336
-7115	9105	0.58
-7114	9104	0.818
-7113	9103	0.688
-7112	9102	0.632
-7111	9101	0.722
-7110	9100	0.762

The sub-samples were cut to be c. 5 mm thick relative to the transverse section. The upper and lower surface were lightly sanded, preserving original pin-holes, but removing any surface contamination and excess resins which come up to the sample surface over time. The upper, lower and radial surfaces were clearly marked with the pin-holes and measurements re-checked prior to dissection. Dissection was carried out using the two versions of the flating astronomical calendar (AD/BC with 0) scale and carried out at times 20 magnification using a binocular microscope and steel disecting blade. The same blade was used to remove any residual contamination possibilities (pinholes, any residual cells from the previously dissected year of interest) prior to sampling each seperate year of interest. Samples were dissected working from youngest to oldest and packed in pre-marked foil packets.

For both time periods covered by this study, the tree-rings were very narrow and the wood highly resinous making dissection a challenge.

Siberian Larch

Tree rings for radiocarbon analysis of Siberian Larch (*Larix sibirica* Ledeb.) were taken from the sample L4756. This sample was collected on 14 August 2019 from a subfossil stem buried in the alluvial deposits of the Tanlova River (Fig. S1.5) in the southern part of the Yamal Peninsula, which is located in the continuous permafrost zone. Most of subfossil wood found in the area grew near the recent polar tree line. Sample L4756 was found 15 km north of the northernmost currently growing trees. The coordinates of the finding location are 67.75 N 70.06 E, 30 m above sea level.

The sample L4756 contained 302 annual rings, which were dated using a 8768 years long Yamal master chronology[13] (Fig. S1.6).

Sample L4756, like all the others subfossil samples collected in Yamal, is archived at the Institute of Plant and Animal Ecology, Ural Branch of the Russian Academy of Sciences, Ekaterinburg, Russia.

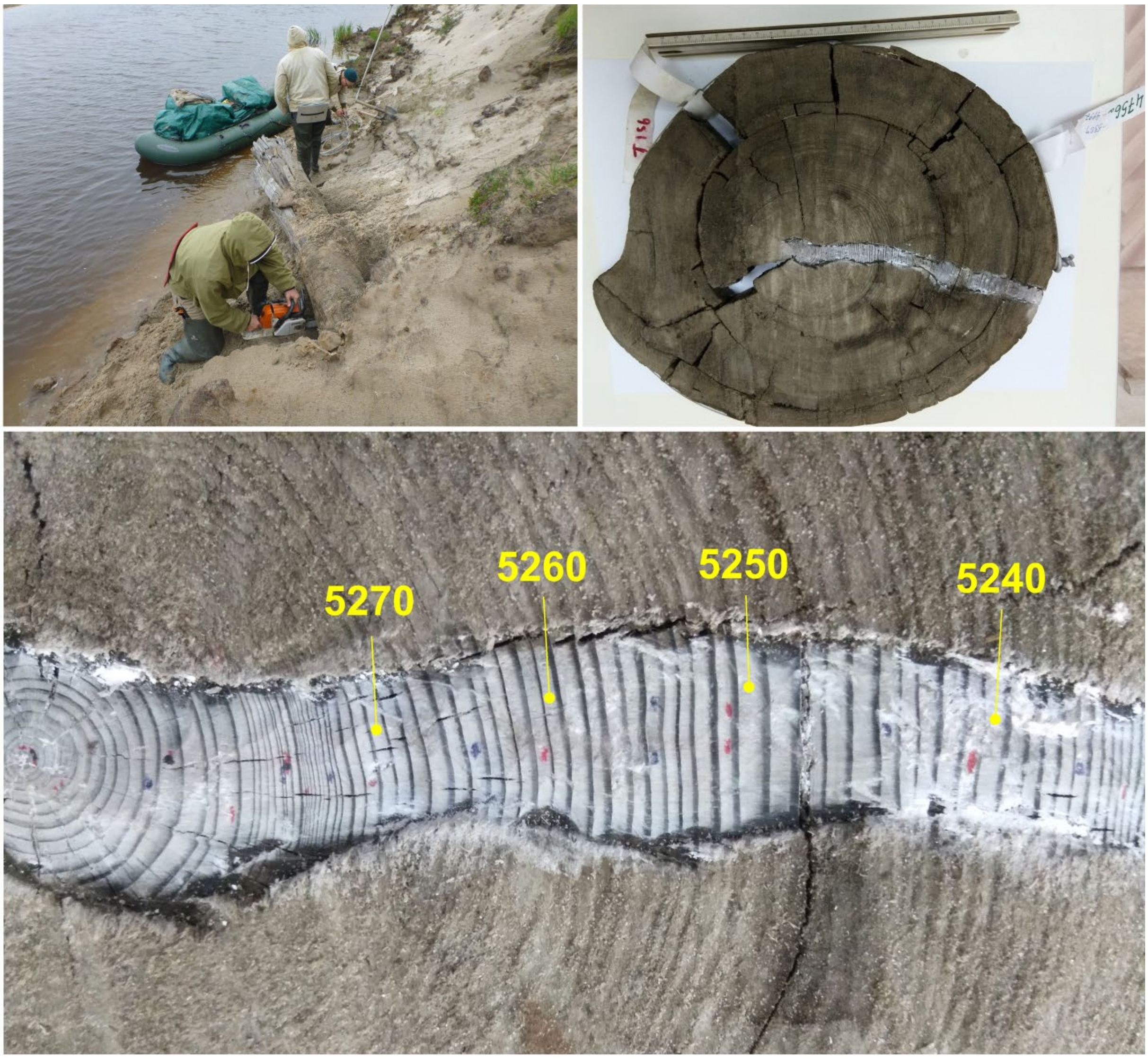

Figure S1.5. Siberian larch used in this work: a) collecting a sample L4756 (Photo by P.Fonti); b) sample L4756; c) tree rings used for analysis (5272-5247 BCE).

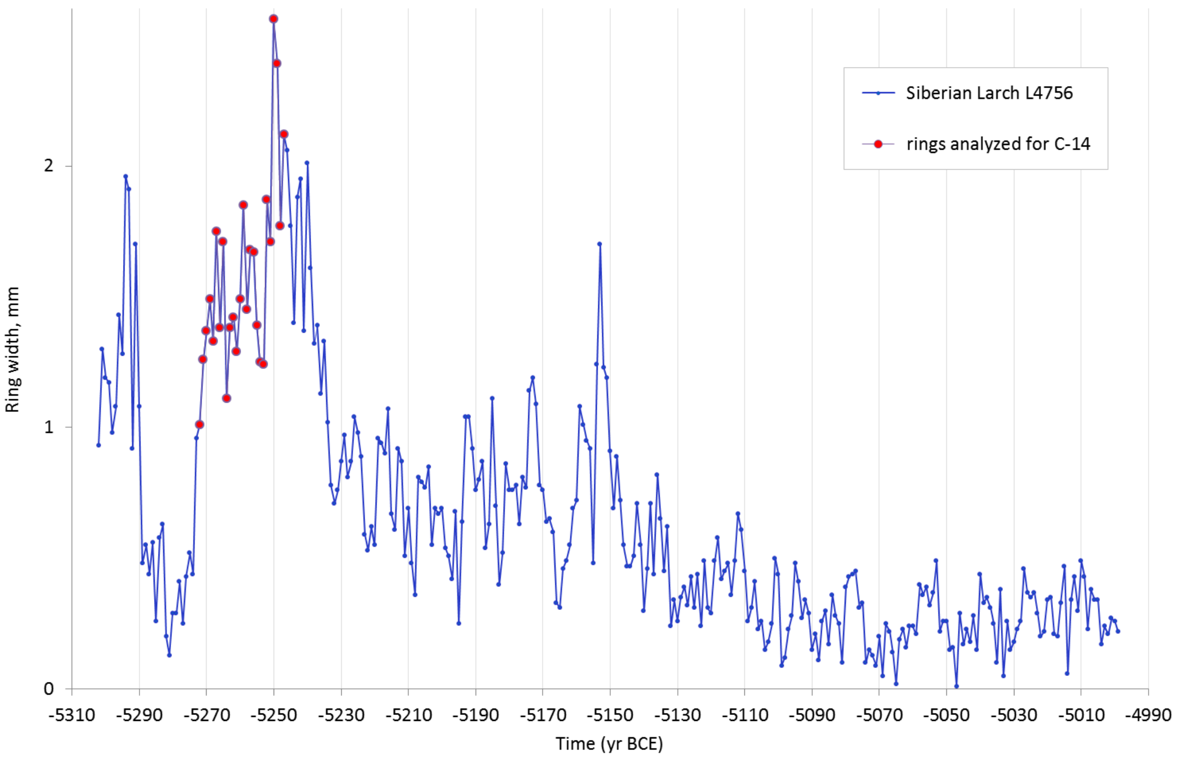

Figure S1.6. Ring width measurements of sample L4756

Dendrochronological dating of two larch samples from the Swiss Alps (ua-1601, tsc-182)

Two larch (*Larix decidua* Mill.) specimens sampled at Alpine glacier forefields were selected for the yearly resolved ^{14}C analyses. The main selection criterion was comparatively wide tree rings.

tsc-182 Alpine Larch

Specimen tsc-182 was collected from a larch log which was uncovered by the retreating Tschierva glacier (46°23' N, 9°52' E), Switzerland, and subsequently displaced by the glacier creek. Field work for sampling was carried out on 15.9.2007. On the specimen tsc-182, 12 (partial) radii were measured with a resolution of 1/1000 mm and subsequently averaged into a 431-year tree-ring width series. The last 54 rings of this series are slightly compressed due to the former covering by the glacier. According to the crossdating (Tab. S1.7) to the Eastern Alpine Conifer Chronology[14] (Fig. S1.7) the 431-year long series (Tab. S1.8) yielded the calendar dating 5337 – 4907 BCE (historical timescale, without year 0).

ua-1601 Alpine Larch

This larch specimen comes from a detrital log found on the forefield of the Unteraar glacier (46°34' N, 8°12' E), Switzerland. The log was sampled in 2016. Four (partial) radii were measured on the specimen with a resolution of 1/1000 mm and subsequently averaged to a 119-year tree-ring width series. The outermost 18 rings of specimen ua-1601 are slightly compressed. This series was crossdated (for statistics see Tab. S1.7) to the Eastern Alpine Conifer Chronology[14,15] (Fig. S1.7) and returned the calendar dates 7242 – 7124 BCE (Tab. S1.8) (historical timescale, without year 0).

Both specimens are stored in the tree-ring sample archive at the Department of Geography, Universität Innsbruck, Austria. The dissection of the wood samples into single tree-rings for the yearly resolved ^{14}C measurements was carried out under a microscope using razor blades.

Table S1.7 *Crossdating statistics for the comparison of the two Alpine larch series tsc-182 and ua-1601 with the Eastern Alpine Conifer Chronology (EACC, Nicolussi et al. 2009, 2015). Calculations were carried out by using the program WinTSAP. Overlap: no. of years; Glk.: Gleichläufigkeit; Sign. Glk.: pointer interval Gleichläufigkeit; t-value$_{BP}$ and t-value$_{H}$: t-values after Baillie and Pilcher as well as Hollstein; dating: historical timescale, without year 0.*

Sample	Reference chronology	Overlap [n]	Glk. (%)	Sign. Glk. [%]	t-value$_{BP}$	t-value$_{H}$	Dating [year BCE]
tsc-182	EACC	431	63	66	7.5	7.4	5337 - 4907
ua-1601	EACC	119	73	76	5.8	7.0	7242 - 7124

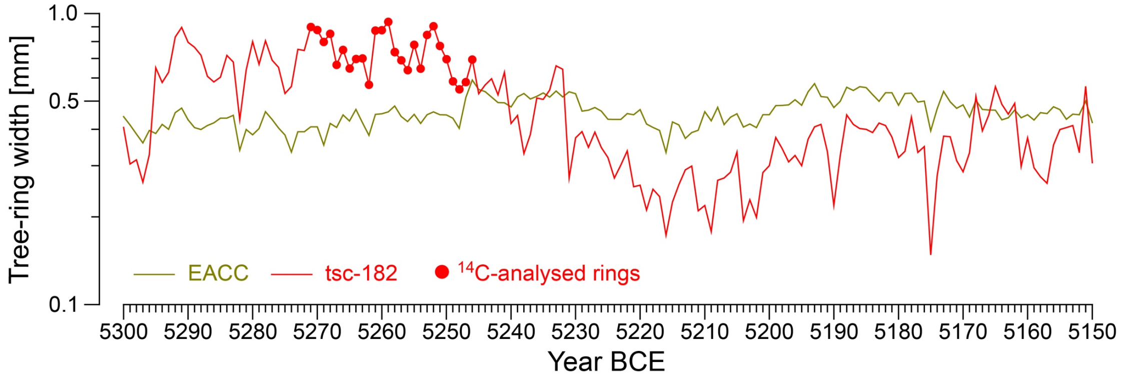

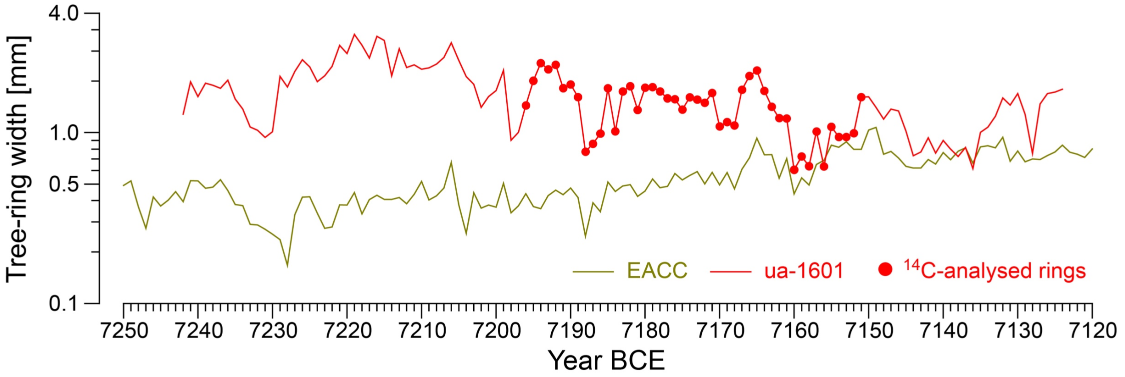

Figure S1.7 *Comparison of the tree-ring width series of the Alpine larch specimens' tsc-182 (upper) and ua-1601 (lower) with the Eastern Alpine Conifer Chronology. The series are given on a logarithmic scale, the tree rings used for single-year ^{14}C analyses are indicated. For ua-1601, a 150-year long section of the series around the ^{14}C-anaysed samples is shown.*

Tab. S1.8 *Tree-ring width series of the two Alpine larch specimen tsc-182 and ua-1601 which provided single-ring samples for radiocarbon analyses (used format: Heidelberg).*

HEADER:
KeyCode=tsc-182
Location=Tschierva glacier
Species=Larix decidua
DataFormat=Tree
SeriesType=Mean curve
Unit=1/1000 mm
Pith=P
WaldKante=---
Length=431

DateBegin=-5337
DateEnd=-4907
Dated=Dated
DATA:Single
86 160 92 71 71 81 48 98 60 61
35 48 40 36 39 80 101 65 53 84
133 132 224 172 214 237 152 151 134 164
213 214 257 170 156 285 374 406 304 314
263 327 651 579 628 830 895 796 765 719
608 582 604 717 681 431 639 800 669 805
691 653 531 561 753 746 899 877 799 852
666 750 647 698 701 569 874 876 936 737
690 639 781 646 844 904 774 697 585 549
581 695 530 568 597 525 628 419 446 330
384 512 507 545 661 643 272 374 393 347
391 346 320 272 301 337 254 257 211 249
235 173 225 258 290 299 210 219 178 267
272 285 334 194 229 199 284 300 375 343
309 325 300 370 408 416 335 219 325 448
416 402 400 390 420 412 376 320 336 440
333 349 148 277 379 377 312 286 332 519
395 448 560 487 451 491 298 374 295 275
261 353 399 405 412 334 562 307 326 417
456 536 428 330 269 128 288 163 215 192
194 234 245 262 330 283 217 250 307 310
282 254 341 547 353 547 427 291 218 252
327 332 381 367 409 252 301 397 356 339
274 326 474 382 450 389 551 469 363 372
396 304 240 261 226 180 219 259 241 283
223 274 212 363 274 324 285 299 266 218
202 283 329 347 431 368 353 376 494 493
398 321 368 313 317 358 388 464 402 367
392 367 322 260 227 231 199 186 178 189
165 196 264 326 360 359 430 529 690 419
291 309 484 559 373 411 420 520 401 492
458 559 556 636 508 459 380 419 577 384
298 420 591 367 401 330 339 273 257 322
272 320 503 482 284 358 288 244 285 281
269 143 142 320 306 227 190 168 124 89
155 199 202 313 247 299 313 345 364 390
358 344 379 348 377 221 176 332 291 308
306 376 479 402 278 304 228 248 197 240
273 334 264 188 222 164 236 227 231 219
262 276 280 240 253 311 321 238 301 269
265 269 251 231 237 262 262 269 286 366
294 278 264 221 271 242 215 120 160 179
178 229 159 112 190 156 188 194 156 140
151

HEADER:
KeyCode=ua-1601
Location=Unteraar glacier
Species=Larix decidua

DataFormat=Tree
SeriesType=Mean curve
Unit=1/1000 mm
Pith=-
WaldKante=---
Length=119
DateBegin=-7242
DateEnd=-7124
Dated=Dated
DATA:Single
1281 1979 1623 1946 1894 1815 2029 1571 1373 1079
1032 938 1011 2134 1850 2278 2672 2420 1986 2152
2443 3239 2903 3754 3251 2733 3659 3457 2152 3090
2403 2494 2351 2393 2515 2750 3355 2643 2126 1916
1404 1625 1761 2313 902 1003 1443 2011 2553 2345
2493 1821 1913 1614 773 861 986 1817 1018 1741
1868 1358 1831 1848 1740 1587 1569 1364 1610 1560
1495 1708 1087 1152 1101 1782 2145 2309 1754 1418
1216 1210 606 725 636 1016 633 1081 943 943
991 1613 1619 1403 1200 1372 1337 1022 732 770
931 758 899 800 725 819 615 1004 1074 1246
1595 1449 1692 1277 754 1469 1692 1726 1796

Supplementary Figures

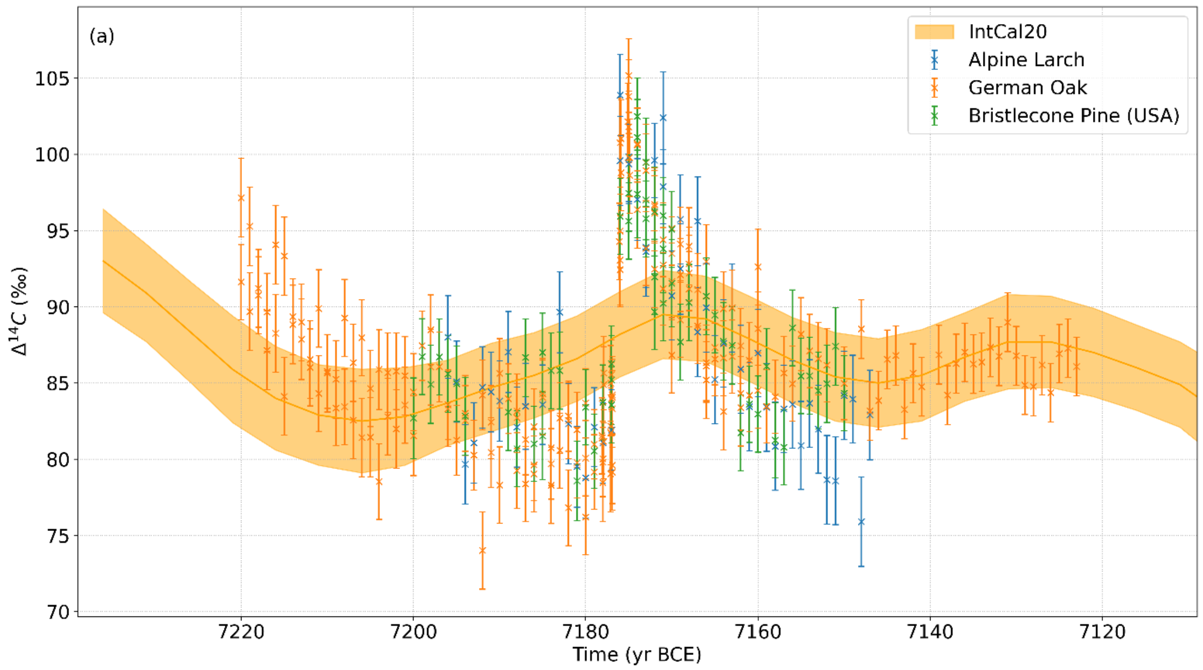

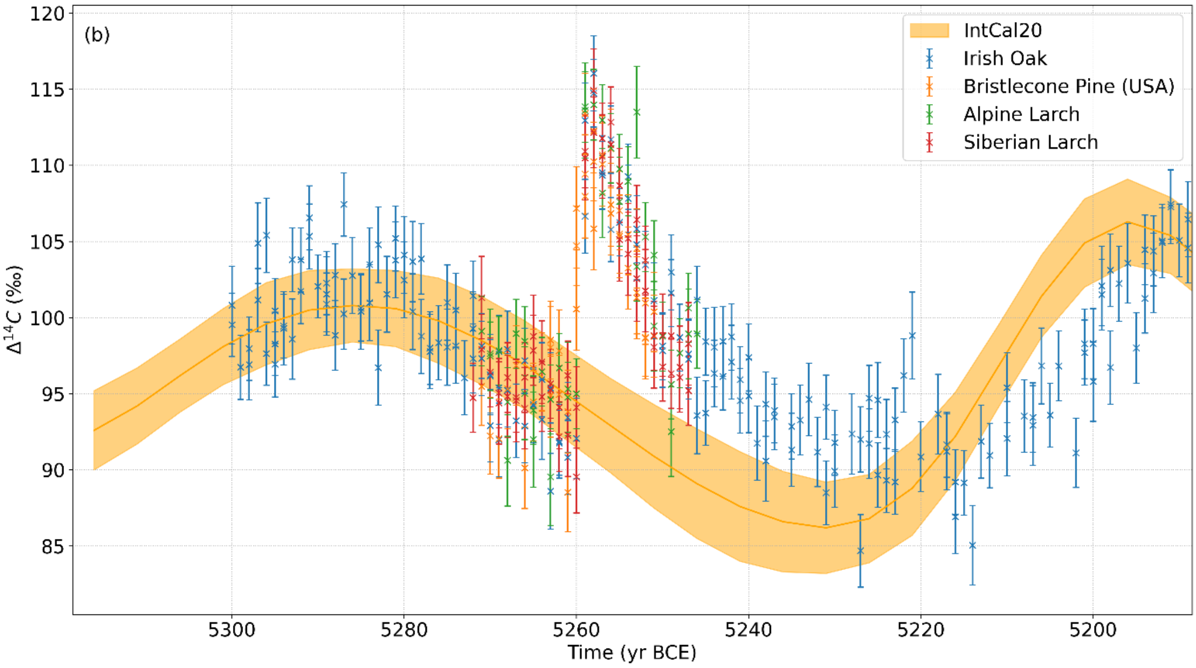

Supplementary Fig. 1 All Measurement results of the 2 new events. *^{14}C measurements with 1-σ errors reported as $\Delta^{14}C$ of the two newly found events (7176 BCE (a), 5259 BCE (b)) in all different trees compared to the IntCal20 calibration curve[16] (orange band). The Irish Oak was independently repeated by two different labs (ETH-Zurich, Bristol).*

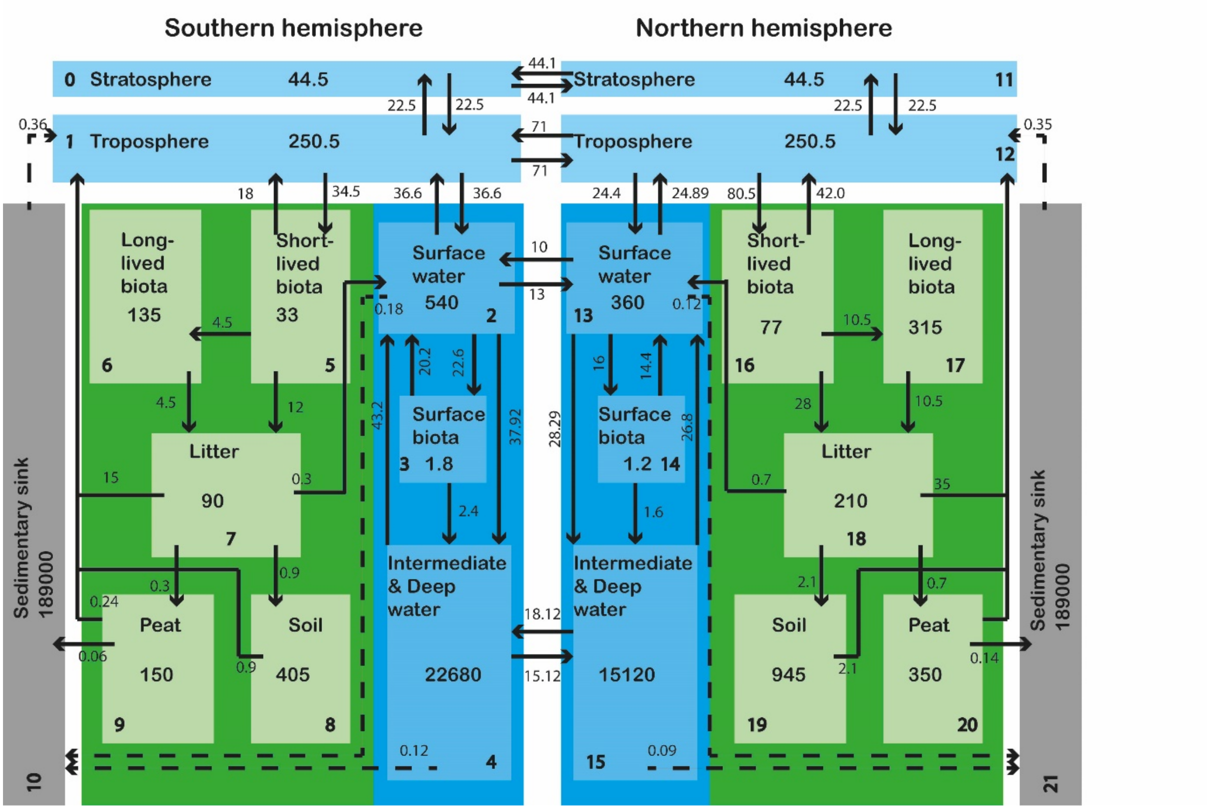

Supplementary Fig. 2 Carbon box model used to reconstruct ^{14}C production. *The carbon fluxes between boxes and their carbon contents are given in Gt/yr and Gt.*

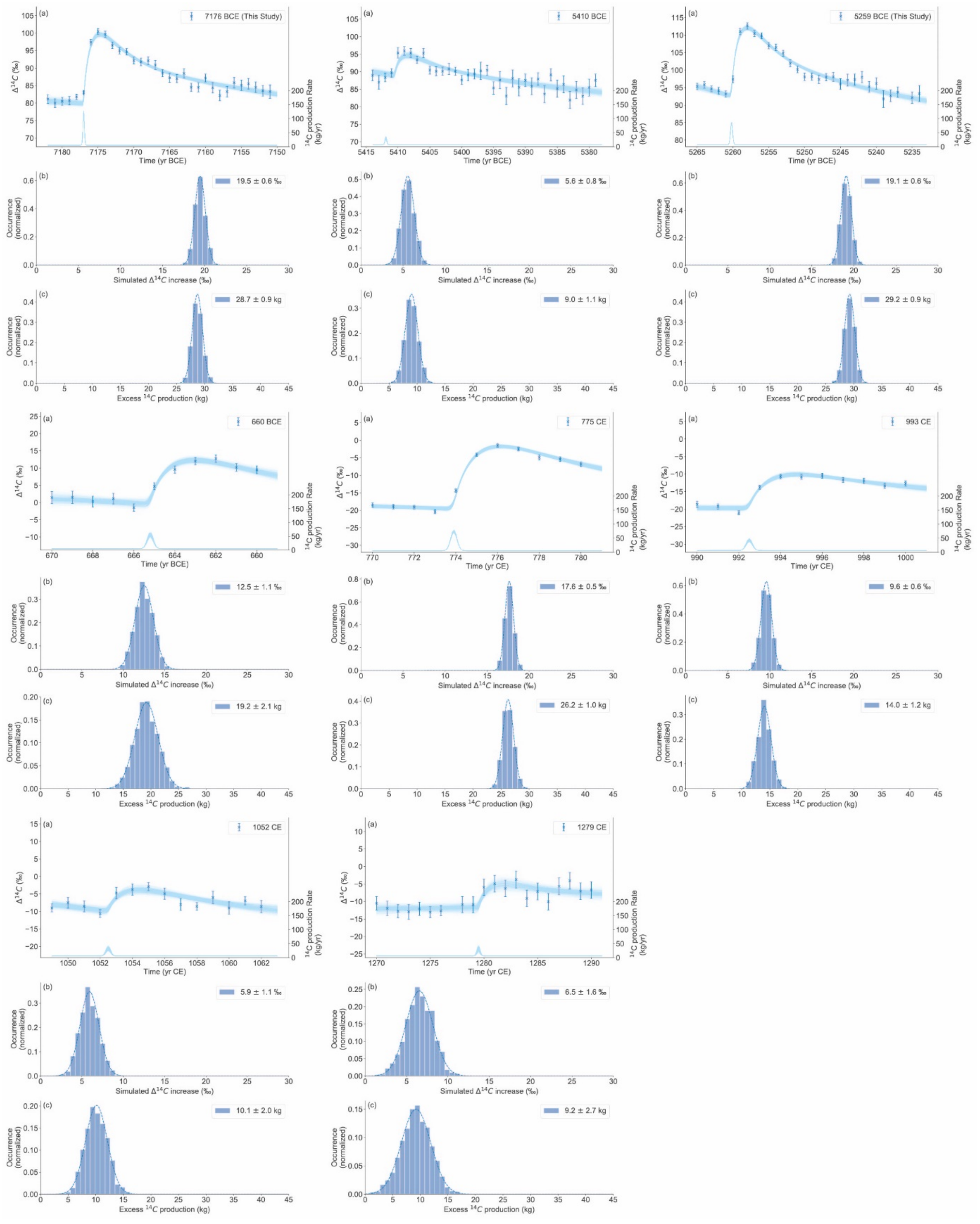

Supplementary Fig. 3 Evaluation of all known ^{14}C events. *(a) Mean data of known ^{14}C events with 1-σ errors and result of 1000 Simulations. The fitted Gaussian shaped production spikes for all simulations are also shown. (b) Distribution of the simulated $\Delta^{14}C$ increases (blue bars) with a Gaussian fit (dashed line). (c) Distribution of excess ^{14}C production (blue bars) with Gaussian fit (dashed line).Data Sources: 5410 BCE: Miyake et al.[17], 660 BCE: Sakurai et al.[18], 775 CE and 993 CE: Büntgen et al.[19], 1052 CE and 1279 CE: Brehm et al.[20].*

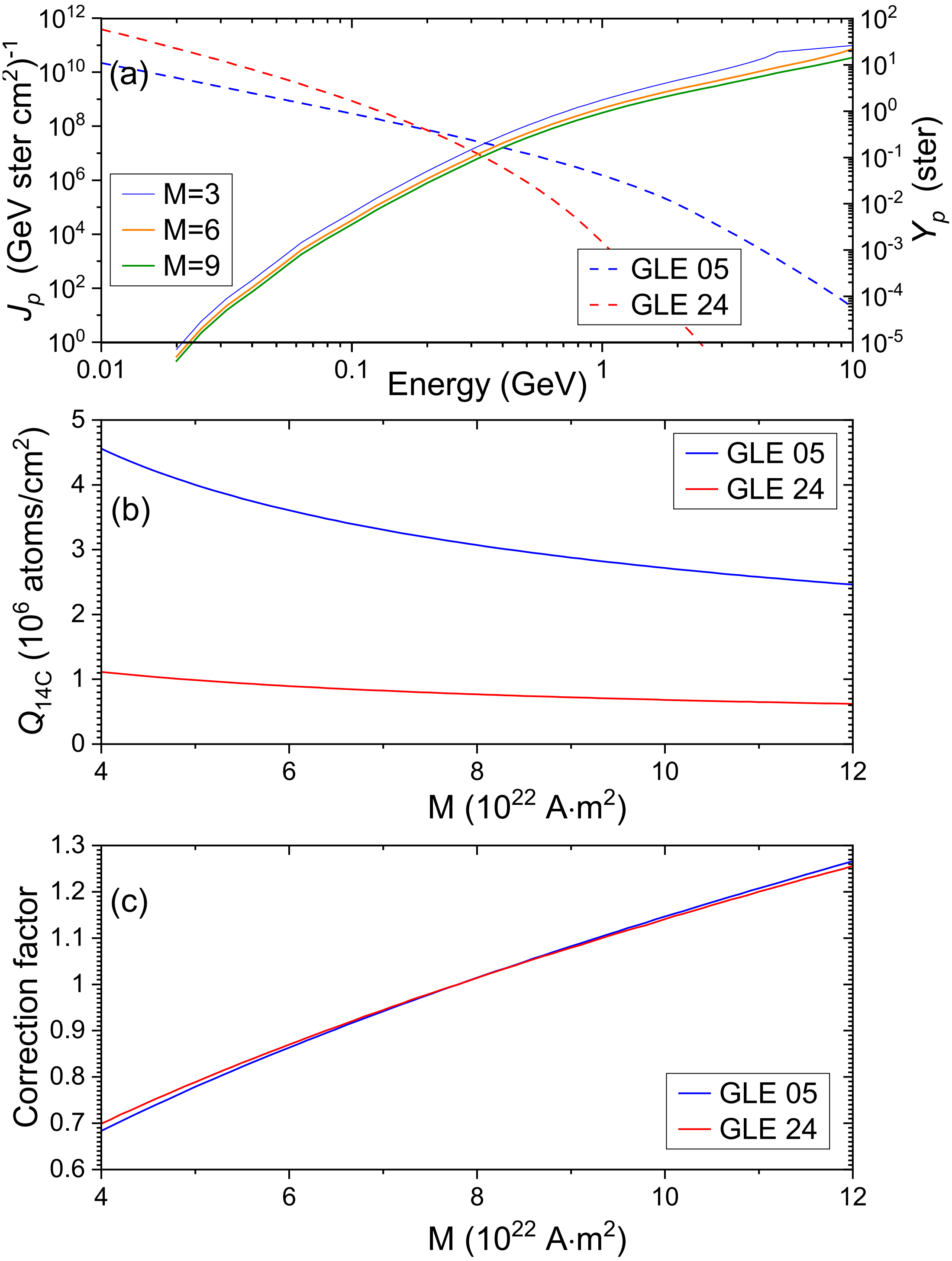

Supplementary Fig. 4 *Production of ^{14}C by SEP events for different values of VADM: (a) Global yield functions (Y_p, right-hand-side axis) of ^{14}C by protons for three values of VADM (3x, 6x and 9x10^{22} A m^2 as blue, orange and green solid lines, respectively); as well as spectral omnidirectional fluences (J(E), left-hand-side axis) of SEPs for two bounding cases, the softest and hardest-spectrum known events of 23-Feb-1956 (GLE 05, blue dashed line) and 04-Aug-1972 (GLE 24, red dashed line). (b) Calculated globally-averaged ^{14}C production Q_{14C} during the two SEP events as a function of the geomagnetic field VADM. (c) Correction factor $Q(M_o)/Q(M)$ of the ^{14}C production at the VADM value of M to that for a modern geomagnetic field (M_o=7.8·10^{22} A m^2).*

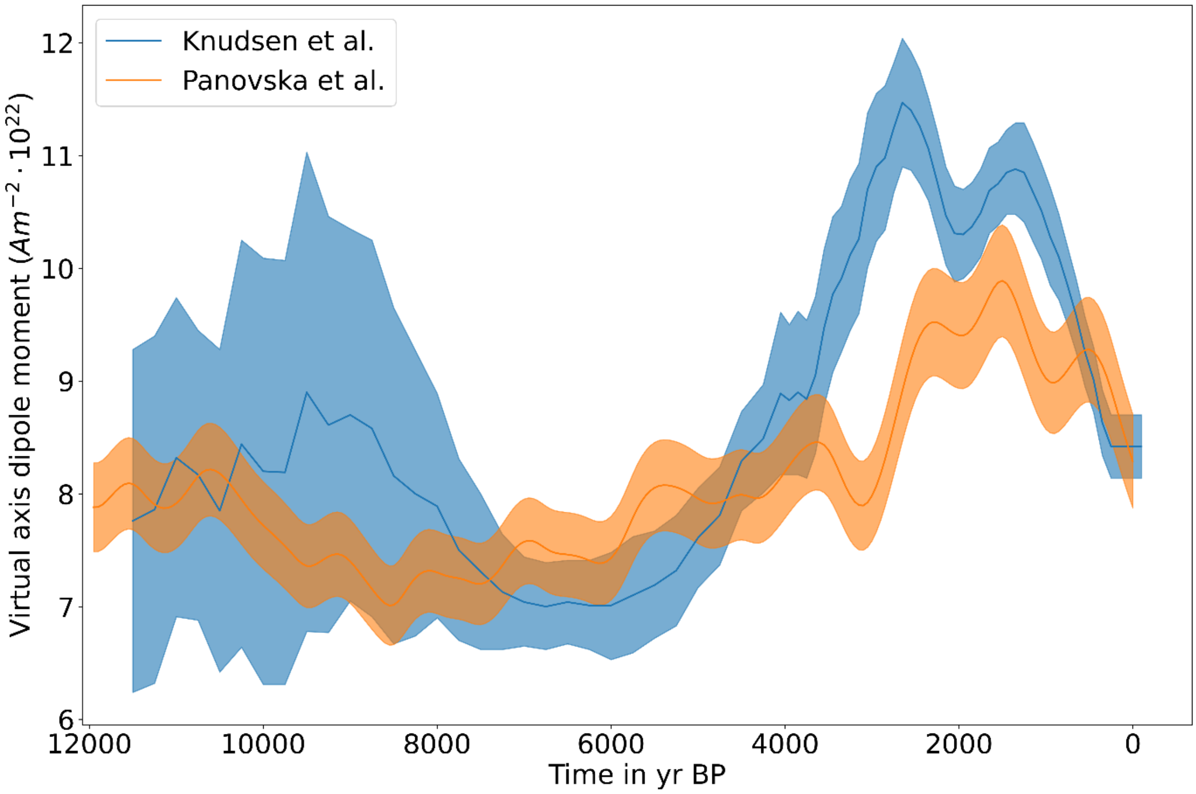

Supplementary Fig. 5 Comparison of two geomagnetic field reconstructions. *Two geomagnetic field reconstructions by Knudsen et al.[21] (blue) and Panovska et al.[22] (orange) over the last 12000 years including 1-σ uncertainty ranges.*

References

1 Baillie, M. G. L. *Tree-ring dating and archaeology*. (London : Croom Helm, 1982).

2 Munro, M. A. R. An Improved Algorithm for Crossdating Tree-Ring Series. (1984).

3 Ferguson, C. W. A 7104-Year Annual Tree-Ring Chronology for Bristlecone Pine, Pinus Aristata, from the White Mountains, California. *Tree-ring Bulletin* (1969).

4 Stokes, M. A. & Smiley, T. L. *An Introduction to Tree-ring Dating*. (University of Arizona Press, 1996).

5 Holmes, R. Computer-Assisted Quality Control in Tree-Ring Dating and Measurement. *Tree-ring Bulletin* (1983).

6 LaMarche Jr., V. C. & Harlan, T. P. Accuracy of tree ring dating of bristlecone pine for calibration of the radiocarbon time scale. *Journal of Geophysical Research (1896-1977)* **78**, 8849-8858, doi:10.1029/JC078i036p08849 (1973).

7 Salzer, M. W. & Hughes, M. K. Bristlecone pine tree rings and volcanic eruptions over the last 5000 yr. *Quaternary Res* **67**, 57-68, doi:10.1016/j.yqres.2006.07.004 (2007).

8 Salzer, M. W. & Hughes, M. K. in *Advances in Global Change Research Advances in Global Change Research* 469-482 (Springer International Publishing, 2010).

9 Salzer, M., Hughes, M., Bunn, A. & Kipfmueller, K. Recent unprecedented tree-ring growth in bristlecone pine at the highest elevations and possible causes. *Proceedings of the National Academy of Sciences of the United States of America* **106**, 20348-20353, doi:10.1073/pnas.0903029106 (2009).

10 Salzer, M. W., Bunn, A. G., Graham, N. E. & Hughes, M. K. Five millennia of paleotemperature from tree-rings in the Great Basin, USA. *Climate Dynamics* **42**, 1517-1526, doi:10.1007/s00382-013-1911-9 (2014).

11 Kipfmueller, K. F. & Salzer, M. W. Linear trend and climate response of five-needle pines in the western United States related to treeline proximity. *Canadian Journal of Forest Research* **40**, 134-142, doi:10.1139/X09-187 (2010).
12 Salzer, M. W., Pearson, C. L. & Baisan, C. H. Dating the Methuselah Walk Bristlecone Pine Floating Chronologies. *Tree-Ring Research* **75**, 61-66, doi:10.3959/1536-1098-75.1.61 (2019).
13 Hantemirov, R. M. *et al.* An 8768-year Yamal Tree-ring Chronology as a Tool for Paleoecological Reconstructions. *Russian Journal of Ecology* **52**, 419-427, doi:10.1134/S1067413621050088 (2021).
14 Nicolussi, K. *et al.* A 9111 year long conifer tree-ring chronology for the European Alps: a base for environmental and climatic investigations. *The Holocene* **19**, 909-920, doi:10.1177/0959683609336565 (2009).
15 Nicolussi, K., Weber, G., Patzelt, G. & Thurner, A. A question of time: extension of the Eastern Alpine Conifer Chronology back to 10071 b2k. *GFZ Potsdam, Scientific Technical Report STR15, Potsdam*, 69-73 (2015).
16 Reimer, P. J. *et al.* The IntCal20 Northern Hemisphere Radiocarbon Age Calibration Curve (0–55 cal kBP). *Radiocarbon* **62**, 725-757, doi:10.1017/RDC.2020.41 (2020).
17 Miyake, F. *et al.* A Single-Year Cosmic Ray Event at 5410 BCE Registered in 14C of Tree Rings. *Geophys Res Lett* **48**, e2021GL093419, doi:10.1029/2021GL093419 (2021).
18 Sakurai, H. *et al.* Prolonged production of 14C during the ~660 BCE solar proton event from Japanese tree rings. *Scientific Reports* **10**, 660, doi:10.1038/s41598-019-57273-2 (2020).
19 Büntgen, U. *et al.* Tree rings reveal globally coherent signature of cosmogenic radiocarbon events in 774 and 993 CE. *Nature Communications* **9**, doi:10.1038/s41467-018-06036-0 (2018).
20 Brehm, N. *et al.* Eleven-year solar cycles over the last millennium revealed by radiocarbon in tree rings. *Nature Geoscience* **14**, 10-15, doi:10.1038/s41561-020-00674-0 (2021).
21 Knudsen, M. F. *et al.* Variations in the geomagnetic dipole moment during the Holocene and the past 50 kyr. *Earth and Planetary Science Letters* **272**, 319-329, doi:10.1016/j.epsl.2008.04.048 (2008).
22 Panovska, S., Constable, C. G. & Korte, M. Extending Global Continuous Geomagnetic Field Reconstructions on Timescales Beyond Human Civilization. *Geochemistry, Geophysics, Geosystems* **19**, 4757-4772, doi:10.1029/2018GC007966 (2018).
